# A Comparison of Different Algorithms for the Assessment of Cardiovascular Risk in Patients at Waiting List for Kidney Transplantation

**DOI:** 10.1371/journal.pone.0161927

**Published:** 2016-10-21

**Authors:** Stefan Reuter, Stefanie Reiermann, Viola Malyar, Katharina Schütte-Nütgen, Renè Schmidt, Hermann Pavenstädt, Holger Reinecke, Barbara Suwelack

**Affiliations:** 1 Department of Internal Medicine D, University Hospital Münster, Münster, Germany; 2 Division of Vascular Medicine, Department of Cardiovascular Medicine, University Hospital Münster, Münster, Germany; 3 Institute of Biostatistics and Clinical Research, University Hospital Münster, Münster, Germany; Robert Bosch Krankenhaus, GERMANY

## Abstract

**Background:**

Cardiovascular disease (CVD) is the leading cause of death after renal transplantation with a high prevalence in dialysis patients. It is still a matter of debate how to assess the cardiovascular risk in kidney transplant candidates. Several approaches and scores exist and found their way into the guidelines.

**Methods and Results:**

We herein assessed PROCAM, Framingham, ESC-SCORE and our own dedicated algorithm in patients applying for renal transplantation at our transplantation center between July 2006 and August 2009. Data of 347 consecutive patients were recorded at baseline and during a follow-up of 4.1 years regarding cardiovascular (CV) events and event-free and overall survival. During follow-up 31 (8.9%) patients died, 24 (6.9%) myocardial infarctions occurred and 19 (5.5%) patients received a new diagnosis of cerebrovascular disease. Predictors for event-free survival identified by univariable Cox regression analysis were age at start of dialysis, ESC-SCORE as well as our own score. Final multivariable model with a stepwise model building procedure revealed age at start of dialysis and smoking to be prognostic for event-free (hazard ratio 1.07/year and 2.15) and overall survival (1.10/year and 3.72).

**Conclusion:**

Comparison of CV risk assessment scores showed that ESC-SCORE most robustly predicted event-free and overall survival in our cohort. We conclude that CV risk assessment by ESC-SCORE can be reasonably performed in kidney transplant candidates.

## Introduction

Chronic kidney disease (CKD) is a leading cause of cardiovascular disease (CVD). In consequence the prevalence of coronary artery disease (CAD) in patients on dialysis is high [1]. Patients with CKD and CAD are more likely to present with an index myocardial infarction than patients without CKD [2]. The mortality of patients with CKD suffering from acute myocardial infarction is considerably high [3–6].

Therefore, patients on the kidney transplant waiting list need a careful evaluation with regard to their individual CV risk burden in order to reduce their perioperative and postoperative risk [7].

These 30–50% of all renal transplant candidates with coronary lesions amenable to revascularization need to be identified, as interventional therapy has been shown to improve their survival [8,9]. It is still a matter of debate how to assess the CV risk in kidney transplant candidates and to identify patients who may benefit from further testing or a coronary intervention prior to transplantation [10,11]. Cumulative 3-year incidence of myocardial infarction ranged from 8.7% to 16.7% after wait listing and from 4.7% to 11.1% after transplantation [11]. In this regard, it was even suggested to perform pre-emptive coronary angiography in transplant (waiting list) patients [8]. CVD remains the leading cause of death after transplantation but it is noteworthy that CV events of renal patients cannot *per se* be accounted to occlusive CAD. Besides CAD, congestive heart failure and fatal ventricular arrhythmias are major causes of cardiovascular morbidity and mortality [12,13]. Thus, simple identification of patients with occlusive CAD may not necessarily identify patients at high risk of a CVD event after renal transplantation. Moreover, coronary angiography is an invasive procedure and cannot be performed regularly and repeatedly in patients on the waiting list. Consequently, a non-invasive approach using a risk stratification score of waiting list candidates is desirable. As many interacting factors including age, gender, smoking, diabetes, arterial hypertension, kidney function etc. may contribute to the individual CV risk statistical approaches have been developed to allow the assessment of patient`s global CV risk in daily clinical practice. In this regard, we performed a retrospectively observational study, assessed and compared the performance for individual CV risk estimation of the standard point-scoring systems PROCAM, Framingham, ESC-SCORE, and our own algorithms (Muenster Cardiovascular Risk Stratification Score) in all newly seen waiting list candidates at our transplantation center between July 2006 and August 2009 [14,15].

## Materials and Methods

### Subjects

We conducted a retrospective study including all adult patients applying for renal transplantation at our transplantation center from July 2006 up to August 2009; patients were followed with a median follow up time of 4.1 years. Patients after heart transplantation, with psychiatric disorders as well as pregnant or breast feeding women were excluded. The following data was collected from patient files: age, gender, body mass index, smoking status, diabetes, blood pressure, lipid profile, c-reactive protein, fibrinogen, start of renal replacement therapy, dialysis modality, renal disease, history of CVD and heart failure, and CV events. Data of all patients were anonymized and de-identified prior to analysis. Written informed consent was given by all participants at the time of listing evaluation for recording their data. Our study was approved by the local ethics committee (Ethik Kommission der Ärztekammer Westfalen-Lippe und der Medizinischen Fakultät der Westfälischen Wilhelms-Universität, No. 2009-461-f-S).

### Scores

Full details of the compared CV-risk assessment scores have been published previously [14,16–18]. S1 Table provides an overview of the parameters used for risk calculation by the scores. In short, PROCAM score-based CV risk estimation includes age, lipids (low-density and high-density lipoprotein cholesterol, triglycerides), smoking, diabetes, family history of CAD, and systolic blood pressure. The Framingham score does not consider family history, diabetes, triglycerides, or differentiation of cholesterol. ESC-SCORE relies on age, gender, systolic blood pressure, smoking habits and total cholesterol. The Muenster Cardiovascular Risk Stratification Score includes information on age, diabetes, history of CAD/cardiac intervention or heart insufficiency. All systems use cardiovascular disease as end point.

### Statistical Analysis

The distribution characteristics of metric variables are described by median values and interquartile ranges (IQR) while categorical data are reported as absolute and relative frequencies. Event-free survival (EFS; death, non-fatal myocardial infarction, new diagnosis of CVD) was calculated as time from start of dialysis up to the time of an event (fatal or non-fatal myocardial infarction or cerebrovascular disease, death from any cause) and overall survival (OS) as time from start of dialysis up to the time of death from any cause. Patients alive without an event were censored at their last follow-up. Kaplan-Meier estimates for EFS and OS were compared by log-rank tests^19^. Cox regression models were applied to analyze the prognostic value of potentially prognostic factors using stepwise variable selection as recommended by Collett [19] (details of model selection are fully described in the Supplementary Methods). Estimated hazard ratios for the selected explanatory variables with their 95% confidence intervals and the associated p-values are given. No adjustment for multiple testing was performed and “significance” refers to local statistical significance defined as a local, unadjusted p-value <0.05. Statistical analyses of retrospective data were performed using SAS software, Version 9.4 for Windows and IBM SPSS Statistics 22 for Windows (IBM Corporation, Somers, NY, USA).

## Results

A total of 347 patients were followed over a median of 4.1 years (IQR 3.1–5.3 yrs.). Baseline characteristics of the study participants are summarized in Table 1. Their median age was 50.6 years, 61.1% were men and >95% were Caucasians. Most patients (91.9%, hemodialysis 78.4%, peritoneal dialysis 13.5%) had been on dialysis for a median time of 11.8 months. The established CV risk factors were highly prevalent: hypertension 95.1%, overweight/obesity 55.3%, dyslipidemia, 61.4%, diabetes 15.5% and active smokers 14.7% (former smokers 16.4%). Besides CKD, overt or a history of CVD were also fairly common: evidence of peripheral arterial disease (PAD) was present in 17.0%, CAD in 15.6% and cerebrovascular disease (CVD) in 13.8%. Stratification of patients into the three risk groups (low, intermediate, high) of the Muenster Cardiovascular Risk Stratification Score is shown in S2 Table. 147 patients have been transplanted during the observation period. Mean waiting was 38.42 months.

**Table 1:** Patients`Demographic Characteristics (n = 347).

		n.o. missings
Age at start of dialysis, year, median (IQR)	50.6 (39.8,58.8)	25* (7.2)
Gender		0
male	212 (61.1)	
female	135 (38.9)	
Body mass index, kg/m^2^, median (IQR)	25.5 (22.9, 28.4)	3 (0.9)
Overweight/obesity		3 (0.9)
yes	192 (55.3)	
no	152 (43.8)	
Blood pressure systolic/diastolic, mm Hg, median (IQR)	133/80 (121/71, 145/85)	24 (6.9)
Smoking		58 (16.7)
never	181 (52.2)	
active smoker	51 (14.7)	
former smoker	57 (16.4)	
Diabetes mellitus		10 (2.9)
no	283 (81.6)	
diabetes mellitus type 1	7 (2.0)	
diabetes mellitus type 2	39 (11.2)	
Diabetes mellitus, other	8 (2.3)	
Hypertension		4 (1.2)
yes	330 (95.1)	
no	13 (3.7)	
Hypercholesterolemia		9 (2.6)
yes	213 (61.4)	
no	125 (36.0)	
Time from first renal replacement therapy	11.8 (5.7, 24.3)	1 (0.3)
to listing, months, median (IQR)		
Dialysis		0
hemodialysis	272 (78.4)	
peritoneal dialysis	47 (13.5)	
no dialysis	28 (8.1)	
Living donation in process		8 (2.3)
yes	54 (15.6)	
no	285 (82.1)	
Medical history		
coronary artery disease or coronary sclerosis	54 (15.6)	
peripheral artery disease	59 (17.0)	
cerebrovascular disease	48 (13.8)	
Renal disease		1 (0.3)
diabetes	24 (6.9)	
glomerulonephritis	79 (22.8)	
hypertension	28 (8.1)	
polycystic kidney disease	51 (14.6)	
unknown	47 (13.5)	
interstitial nephritis	30 (8.6)	
reflux nephropathy	15 (4.3)	
focal segmental sclerosis	17 (4.9)	
vasculitis	13 (3.7)	
other	42 (12.1)	

Absolute and relative frequency (referred to n = 347) for categorial data, median and interquartile range (IQR) for metric data. Overweight/obesity was noted if BMI was ≥ 25 kg/m^2^. Hypertension was recorded in case of antihypertensive medical treatment; hypercholesterinemia if the patient received a statin or total cholesterol was >6.2 mmol/L (240 mg/dL); history of coronary artery disease comprised previous myocardial infarction or proof by angiography; cerebrovascular disease comprised stroke or transitory cerebral ischemia; peripheral artery disease comprised proof of plaque by imaging or verified intermittent claudication.

*25 patients with unknown date of start of dialysis (patients with ESRD but not on dialysis).

### Long-term Outcomes

Table 2 shows that 31 patients (8.9%) died during the follow up (19 before renal transplantation/12 after renal transplantation), 7 due to cardiovascular disease (4/3), 10 of non-CVD (3/7) and for 14 (12/2) the cause was unknown. There were in addition 24 nonfatal myocardial infarctions (16/8) and 19 (15/4) patients had a new diagnosis of CVD on the waiting list.

**Table 2:** Events during follow-up.

		Before Tx (n)	After Tx (n)
Death		19	12
	cardiovascular reason	4	3
	noncardiovascular reason	3	7
	unknown	12	2
Myocardial infarction (non fatal)		16	8
New diagnosis of cerebrovascular disease		14	5

### Comparison of the prediction models

**Fig 1:**
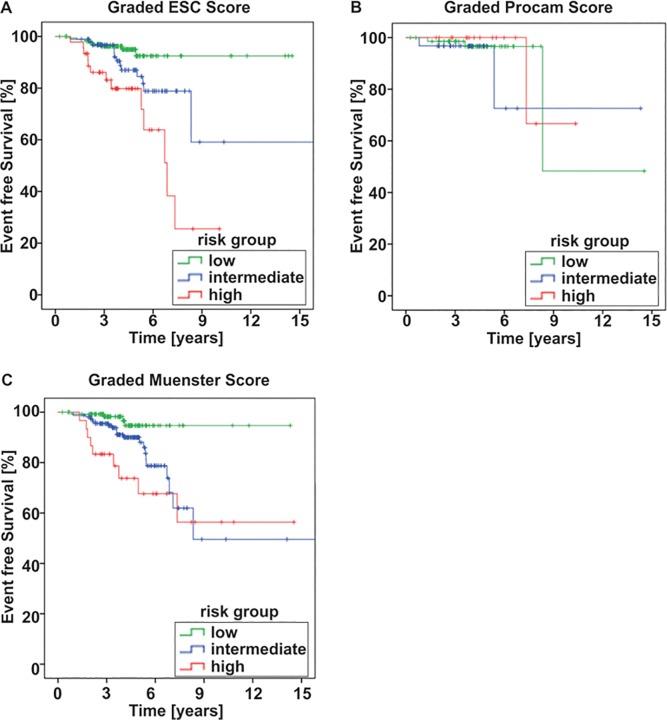
**Event-free survival by graded ESC (A), PROCAM (B) and Muenster (C) score.** Shown are the performances of low, intermediate and high risk groups for each score.

Univariable analysis of predictive factors with Cox regression (Table 3) revealed that age at the start of dialysis was the only factor that was strongly related to event-free survival (p<0.001) as well as overall survival (p<0.001, S3 Table). Moreover, prevalent CAD (p = 0.08) and CVD (p = 0.013) were associated with EFS, but with lower statistical significance, presumably due to low numbers and reduced statistical power. Furthermore, Table 4 shows that univariable regression analysis identified the ESC-SCORE (p ≤0.001) as well as the Muenster Score (p = 0.002) to significantly predict the event-free survival (and, likewise, the overall survival (p<0.001 and p = 0.003, respectively, S4 Table). The PROCAM and the Framingham scores were not predictive among the CKD patients of this study. Fig 1 and S1 Fig display the distributions of EFS and OS by the risk groups.

**Table 3:** Univariable analysis for event-free survival assessing potential risk factors.

Variable	Available cases	HR	95% CI	P
**Gender**	322			**0.469**
**female versus male**	124 vs. 198	0.79	0.41 to 1.50	
**Active smoking**	298			**0.282**
**yes versus no**	48 vs. 250	1.60	0.70 to 3.63	-
**History of smoking**	274			**0.580**
**yes versus no**	50 vs. 224	1.33	0.49 to 3.60	-
**Hypertension**	319			**0.311**
**yes versus no**	307 vs. 12	0.51	0.16 to 1.68	
**Diabetes mellitus**	313			**0.622**
**yes versus no**	51 vs. 262	1.26	0.52 to 3.08	
**Hypercholesterinemia**	314			**0.756**
**yes versus no**	202 vs. 112	1.12	0.54 to 2.31	
**Hyperlipidemia**	318			**0.764**
**yes versus no**	131 vs. 102	1.31	0.58 to 2.94	
**mixed versus no**	85 vs. 102	1.05	0.40 to 2.72	
**Cerebrovascular disease**	317			**0.013**
**yes versus no**	45 vs. 272	2.76	1.31 to 5.79	
**Peripheral artery disease**	313			**0.406**
**yes versus no**	42 vs. 271	1.45	0.62 to 3.39	
**Coronary artery disease**	319			**0.080**
**yes versus no**	53 vs. 266	1.93	0.94 to 3.96	
**Chronic heart insufficiency**	317			**0.380**
**yes versus no**	19 vs. 298	1.58	0.60 to 4.17	
**Age at the start of dialysis**	322	1.07	1.04 to 1.10	**<0.001**
**Body mass index**	319	1.00	0.93 to 1.08	**0.963**
**C-related protein**	289	1.00	0.91 to 1.11	**0.934**
**Fibrinogen**	183	1.002	1.000 to 1.005	**0.099**
**Dialysis time at time of wait listing**	313	1.012	0.996 to 1.027	**0.178**

Estimated hazard ratio (HR) for event-free survival with 95% confidence interval (CI) and p-value of the likelihood ratio test. For pairwise comparisons, confidence intervals instead of p-values are given (p-value of Wald test ≤ 0.05 if and only if confidence interval does not contain 1).

**Table 4:** Univariable analysis for event-free survival assessing different risk stratification scores.

Variable	Available cases	HR	95% CI	P
***D-ESC Score***	309	1.17	1.08 to 1.27	**0.001**
***Graded D-ESC Score***	309			**<0.001**
**intermediate versus low risk**	94 vs. 170	2.37	0.98 to 5.75	
**high versus low risk**	45 vs. 170	6.16	2.55 to 14.91	
***Procam Health Score***	117	1.02	0.93 to 1.11	**0.738**
***Graded Procam Health Score***	117			**0.783**
**intermediate versus low risk**	31 vs. 70	1.84	0.03 to 11.16	
**high versus low risk**	16 vs. 70	0.90	0.09 to 8.87	
***Framingham Score***	118	1.01	0.89 to 1.15	**0.871**
***“Muenster Risk Stratification”***	320			**0.002**
**intermediate versus low risk**	160 vs. 130	3.82	1.32 to 11.11	
**high versus low risk**	30 vs. 130	6.84	2.07 to 22.56	

Estimated hazard ratio (HR) for event-free survival with 95% confidence interval (CI) and p-value of the likelihood ratio test. For pairwise comparisons, confidence intervals instead of p-values are given (p-value of Wald test ≤ 0.05 if and only if confidence interval does not contain 1)

Multivariable models (Table 5), revealed age at start of dialysis to be predictive for event-free survival (hazard ratio 1.07 per year of age, p <0.001); active smoking seemed also related (HR = 2.15, p = 0.09). Similarly, overall survival was also associated with age (HR = 1.10 per year of age;p < 0.001) as was active smoking (HR = 3.72, P = 0.008; Table 6). Comparison of CV risk assessment scores showed that ESC-SCORE most robustly predicted event-free (Fig 1) and overall survival in our population (Table 7).

**Table 5:** Multivariable analysis for event-free survival.

Variable	Available cases	HR	95% CI	P
**Age at start of dialysis**	298	1.07	1.03 to 1.11	**<0.001**
**Active smoking**	298			**0.090**
**yes versus no**	48 vs. 250	2.15	0.93 to 4.98	

Final multivariable model using a stepwise model building procedure. Estimated hazard ratio (HR) with 95% confidence interval (CI) and p-value of the likelihood ratio test. For pairwise comparisons, confidence intervals instead of p-values are given (p-value of Wald test ≤ 0.05 if and only if confidence interval does not contain 1)

**Table 6:** Multivariable analysis for overall survival.

Variable	Available cases	HR	95% CI	P
**Age at start of dialysis**	298	1.10	1.05 to 1.15	**<0.001**
**Active smoking**	298			**0.008**
**yes versus no**	48 vs. 250	3.72	1.50 to 9.23	

Final multivariable model using a stepwise model building procedure. Estimated hazard ratio (HR) with 95% confidence interval (CI) and p-value of the likelihood ratio test. For pairwise comparisons, confidence intervals instead of p-values are given (p-value of Wald test ≤ 0.05 if and only if confidence interval does not contain 1)

**Table 7:** Multivariable analysis for overall survival (comparison of scores).

Variable	Available cases	HR	95% CI	P
***“Muenster Risk Stratification”***	307			**N/S**
**intermediate versus low**	156 vs. 122	--	--	
**high versus low**	29 vs. 122	--	--	
***Graded D-ESC***	307			**N/S**
**intermediate versus low**	92 vs. 170	--	--	
**high versus low**	45 vs. 170	--	--	
***D-ESC Score***	307	1.22	1.12 to 1.33	**<0.001**

Final multivariable model using a stepwise model building procedure. Estimated hazard ratio (HR) with 95% confidence interval (CI) and p-value of the likelihood ratio test. For pairwise comparisons, confidence intervals instead of p-values are given (p-value of Wald test ≤ 0.05 if and only if confidence interval does not contain 1). N/S: not selected

## Discussion

Preemptive assessment of CV-risk is commonly performed before kidney transplantation to identify patients at risk [20–22]. Because this evaluation usually takes place when surgery is still undetermined (for several years) questions arise. For example, transplant centers reevaluate patients on their waiting list frequently. As patients´ health status generally deteriorate while waiting for organ transplantation, the intervals need to be defined and safety as well as efficacy issues need to be considered in this high risk cohort.

For cardiac (re-)evaluation, more aggressive strategies including invasive diagnostic testing for all patients are contrasted by non-invasive test-based approaches or examination according to their symptomatic status. This is important since it has been shown that CV event–free survival is not different between waiting list patients with normal coronary arteries, non-obstructive CAD, or obstructive CAD with intervention, but considerably better than that of patients with obstructive CAD without intervention [8,23].

In the general population, equation-derived estimation of CVD-risk according e.g. to the Framingham study data is established. In order to simplify screening equations-based strategies, a CVD-risk grading or categorization to offer a risk-dependent individualized stratification of transplant candidates for cost-effective evaluation is required. On one hand, approaches should be simple and help to avoid patient`s exposure to unnecessary procedures or testing-derived risks. On the other hand, patients with a high CVD-risk who might benefit from invasive cardiac intervention or strict CVD-risk factor minimization should be detected. According to Kasiske et al. a risk-stratified screening strategy effectively avoids unnecessary testing in more than 40% of the patients [24]. Two examples of recent guidelines recommended risk stratification for CVD screening in transplant candidates are the “European Best Practice Guideline on the Management and Evaluation of the Kidney Donor and Recipient” and the “Cardiac disease evaluation and management among kidney and liver transplantation candidates: a scientific statement from the American Heart Association and the American College of Cardiology Foundation: endorsed by the American Society of Transplant Surgeons, American Society of Transplantation, and National Kidney Foundation”[20,22]. Another option, the current Kidney Health Australia–Caring for Australasians with Renal Impairment Guideline for the assessment of the transplant recipients, provides a simple list of CV-risk factors. According to this guideline, indicators of high risk are older age, diabetes mellitus, abnormal echocardiogram, previous ischemic heart disease or congestive heart failure, increased duration of dialysis or smoking [21]. However, the evidence for the suggested risk classifications is low and all the recommendations should be regarded as an expert opinion [20]. At present clear and uniform guidelines in regards to the CVD-screening (risk-stratification, tests performed, or frequency of reassessment) of waiting list candidates are still missing.

Since Framingham, different point scoring systems for the assessment of CV-risk in the general population have been developed and validated. However, usual CVD-risk factors were very poor predictors of obstructive CAD in kidney transplantation candidates [23]. The particular morbidity, i.e. the special type and burden of atherosclerosis in renal patients, might question the appropriateness of the appraisal CV-risk assessment (scores) to these patients. Therefore, this study was conducted to clarify the role of these scores, like PROCAM, Framingham, ESC-SCORE, or our own algorithm (Muenster Cardiovascular Risk Stratification Score) [14,14,15,20–22]) for CVD-risk stratification of kidney transplant candidates. In our observational, retrospective study we explored the performance of the different CVD-risk assessment scores over a 4-year period in 347 patients with a median follow-up of 4.1 year at a large academic transplant center.

Two important findings derive from our analysis: (1) of all classical CVD-risk factors only age and smoking were predictors of CV events and survival, and (2) ESC-SCORE most robustly predicted event-free and overall survival in renal transplant candidates. ESC-SCORE is a relatively simple (to handle) score using a manageable number of parameters namely age, gender, systolic blood pressure, smoking habits and total cholesterol for risk calculation. Notably, 2 out of these 5 variables were confirmed here as prognostic factors by multivariable analysis with a hazard ratio 1.07/year of age and 2.15 for smoking in regards to event-free survival, and a hazard ratio 1.10/year of age and 3.72 for smoking in regards to overall survival, respectively. In addition to ESC-SCORE, the Muenster score predicted event-free and overall survival with patients in medium and high risk category showing similar event and survival rates. This score stratifies low and medium risk patients according to age (<50 or >50 years) amongst other factors. The median age in the high risk group was 55 years. This is comparable to 56.7 years in the medium risk group whereas the low risk groups had a median age of 39.1 years leaving age to probably be the decisive parameter. The inferior performances of Framingham and PROCAM scores in our patients were related to the inclusion of too many probably in this setting irrelevant or not fate determining factors like sex and blood pressure (both scores), HDL cholesterol (Framingham), diabetes, history of CVD, body weight and height (PROCAM).

## Conclusion

We conclude from our data that age at start of dialysis and smoking are prognostic for event-free and overall survival. In a comparison of CV risk assessment scores ESC-SCORE most robustly predicted event-free and overall survival in our waiting list candidates. Therefore, CV risk stratification by ESC-SCORE can be reasonably performed in kidney transplant candidates. This strategy should be followed in a prospective study.

## Supporting Information

S1 Fig Overall survival by different scores. (TIF)

S1 Supplemental Methods (DOCX)

S1 Table Overview of the parameters used for risk calculation by the different scores. (DOCX)

S2 Table Patients`categorization according to “Muenster cardiovascular Risk Stratification Score”. Absolute and relative frequency for categorial data, median and interquartile range (IQR) for metric data. * missings to add up to 347: Two patients with unavailable categorization according to “Muenster cardiovascular Risk Stratification Score”. ** 16/8/1 patient with unknown date of start of dialysis in the low/intermediate/high risk group (patients with ESRD but not on dialysis).
(DOCX)

S3 Table Univariable analysis for overall survival assessing potential risk factors. Estimated hazard ratio (HR) for overall survival with 95% confidence interval (CI) and p-value of the likelihood ratio test. For pairwise comparisons, confidence intervals instead of p-values are given (p-value of Wald test ≤ 0.05 if and only if confidence interval does not contain 1).
(DOCX)

S4 Table Univariable analysis for overall survival assessing different risk stratification scores. Estimated hazard ratio (HR) for overall survival with 95% confidence interval (CI) and p-value of the likelihood ratio test. For pairwise comparisons, confidence intervals instead of p-values are given (p-value of Wald test ≤ 0.05 if and only if confidence interval does not contain 1).
(DOCX)
