# Supplementary figures and images for: A Comparison of Different Algorithms for the Assessment of Cardiovascular Risk in Patients at Waiting List for Kidney Transplantation
Source: PLoS One. 2016 Oct 21;11(10):e0161927. doi: 10.1371/journal.pone.0161927 (PMC5074508; doi:10.1371/journal.pone.0161927)

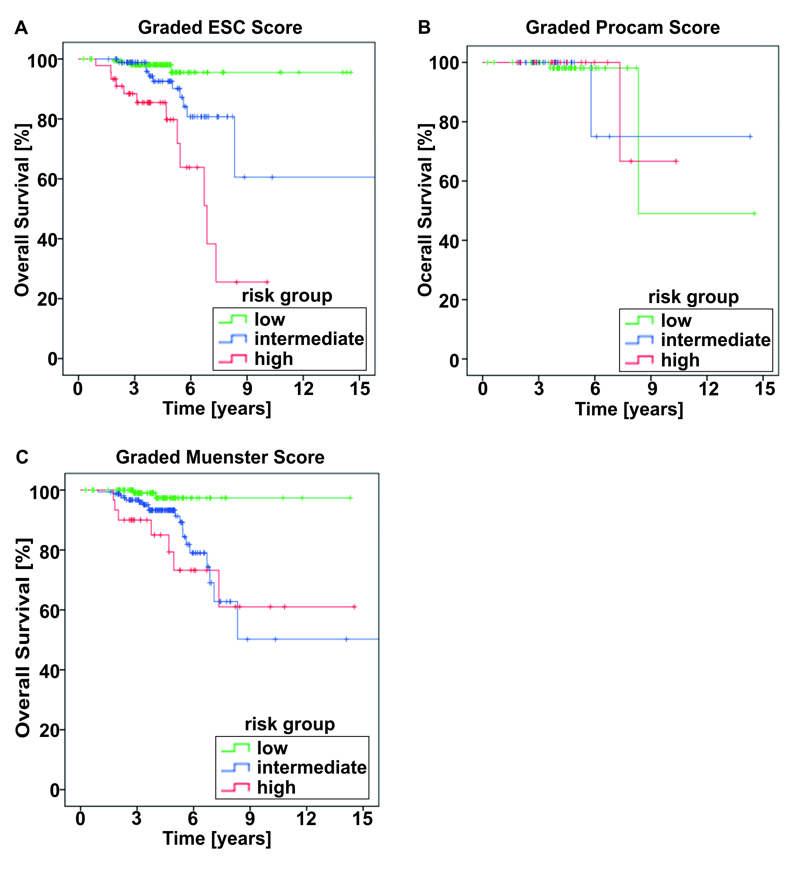

Supplement: S1 Fig — (TIF) [file pone.0161927.s001.tif]
