# Supplementary material for: A Comparison of Different Algorithms for the Assessment of Cardiovascular Risk in Patients at Waiting List for Kidney Transplantation
Source: PLoS One. 2016 Oct 21;11(10):e0161927. doi: 10.1371/journal.pone.0161927 (PMC5074508; doi:10.1371/journal.pone.0161927)
Supplement: S1 Supplemental Methods — (DOCX) [file pone.0161927.s002.docx]

**Supplementary Methods**

**Multivariable Model Building**

Multivariable Cox regressions were used to analyze the prognostic value of the following potentially explanatory prognostic factors with respect to event-free survival (EFS) and overall survival (OS): ESC-SCORE (continuous), graded ESC-SCORE (high v intermediate v low risk) and “Muenster Risk Stratification” (high v intermediate v low risk), as well as the potential risk factors listed in Table 3. Procam Health Score and Framingham Score were not included in multivariable analysis due to high percentage of missing values (66%).

Cox regression models were built following a stepwise variable selection procedure recommended by Collet (19). In the first step, all factors were tested in a univariable Cox regression one at a time. In step 2, all factors that appeared to be important in step 1 (p-value of the likelihood ratio test ≤ .1) were fitted together by a stepwise backward selection of a multivariable Cox regression (p-value of the likelihood ratio test > .1 as exclusion criterion). In step 3, all factors that were not important in step 2 were added to the model of step 2, one at a time, to test their prognostic impact. The Collett procedure stopped at that stage. Finally, the factors of model 3 with all pair-wise interactions were fitted in a stepwise backward selection (p-value of the likelihood ratio test > .1 as exclusion criterion).

In addition, a multivariable Cox regression (stepwise forward selection) was applied for the total cohort, including the three risk scores ESC-SCORE (continuous), graded ESC-SCORE (high v intermediate v low risk as reference) and “Muenster Risk Stratification” (high v intermediate v low risk as reference), in order to compare performance of the risk scores.
