# Supplementary material for: A Comparison of Different Algorithms for the Assessment of Cardiovascular Risk in Patients at Waiting List for Kidney Transplantation
Source: PLoS One. 2016 Oct 21;11(10):e0161927. doi: 10.1371/journal.pone.0161927 (PMC5074508; doi:10.1371/journal.pone.0161927)
Supplement: S1 Table — (DOCX) [file pone.0161927.s003.docx]

**S1 Table.** Overview of the parameters used for risk calculation by the different scores

| **Variable** | **ESC** | **Framingham** | **Muenster** | **Procam** |
| --- | --- | --- | --- | --- |
| **Gender** | X | X |  | X |
| **Age** | X | X | X | X |
| **Smoking** | X | X |  | X |
| **History of CVD** |  |  | X | X |
| **Blood pressure** | X | X |  | X |
| **Diabetes mellitus** |  |  | X | X |
| **Triglycerides** |  |  |  | X |
| **Total cholesterol** | X | X |  |  |
| **HDL cholesterol** |  | X |  | X |
| **Symptoms/physical fitness** |  |  | X |  |
| **Electrocardiogram** |  |  | X |  |
| **Body weight** |  |  |  | X |
| **Height** |  |  |  | X |
