# Supplementary material for: A Comparison of Different Algorithms for the Assessment of Cardiovascular Risk in Patients at Waiting List for Kidney Transplantation
Source: PLoS One. 2016 Oct 21;11(10):e0161927. doi: 10.1371/journal.pone.0161927 (PMC5074508; doi:10.1371/journal.pone.0161927)
Supplement: S2 Table — Absolute and relative frequency for categorial data, median and interquartile range (IQR) for metric data. * missings to add up to 347: Two patients with unavailable categorization according to “Muenster cardiovascular Risk Stratification Score”. ** 16/8/1 patient with unknown date of start of dialysis in the low/intermediate/high risk group (patients with ESRD but not on dialysis). (DOCX) [file pone.0161927.s004.docx]

**S2 Table.**

Patients` categorization according to “Muenster cardiovascular Risk Stratification Score”

|  | **low risk** | **(n=146)*** | n. o. |
| --- | --- | --- | --- |
|  |  |  | missings |
| Age at start of dialysis, year, median (IQR) | | 39.1 (33.3, 44.5) | 16** (11.0) |
| Gender | |  | 0 |
|  | male | 57 (39.0) |  |
|  | female | 89 (61.0) |  |
| Body mass index, kg/m^2^, median (IQR) | | 24.8 (22.6, 27.8) | 1 (0.7) |
| Blood pressure systolic/diastolic, mmHg, median (IQR) | | 132/80 (120/75, 147/90) | 10 (6.8) |
| Smoking | |  | 17 (11.6) |
|  | never | 83 (56.9) |  |
|  | active smoker | 25 (17.1) |  |
|  | former smoker | 21 (14.4) |  |
| Diabetes mellitus | |  | 4 (2.7) |
|  | no | 139 (95.2) |  |
|  | diabetes mellitus type 1 | 2 (1.4) |  |
|  | diabetes mellitus type 2 | 1 (0.7) |  |
|  | diabetes mellitus, other | 0 |  |
| Hypertension | |  | 2 (1.4) |
|  | yes | 137 (93.8) |  |
|  | no | 7 (4.8) |  |
| Hypercholesterolemia | |  | 4 (2.7) |
|  | yes | 70 (48.0) |  |
|  | no | 72 (49.3) |  |
|  | **medium risk** | **(n=168)*** | n. o. |
|  |  |  | missings |
| Age at start of dialysis, year, median (IQR) | | 56.7 (52.2, 64.2) | 8** (4.8) |
| Gender | |  | 0 |
|  | male | 99 (58.9) |  |
|  | female | 69 (41.1) |  |
| Body mass index, kg/m^2^, median (IQR) | | 25.6 (23.0, 28.7) | 1 (0.6) |
| Blood pressure systolic/diastolic, mmHg, median (IQR) | | 131/80 (122/70, 140/83) | 8 (4.8) |
| Smoking | |  | 30 (17.8) |
|  | never | 87 (51.8) |  |
|  | active smoker | 21 (12.5) |  |
|  | former smoker | 30 (17.9) |  |
| Diabetes mellitus | |  | 2 (1.2) |
|  | no | 124 (73.8) |  |
|  | diabetes mellitus type 1 | 3 (1.8) |  |
|  | diabetes mellitus type 2 | 31 (18.4) |  |
|  | diabetes mellitus, other | 8 (4.8) |  |
| Hypertension | |  | 1 (0.6) |
|  | yes | 162 (96.4) |  |
|  | no | 5 (3.0) |  |
| Hypercholesterolemia | |  | 4 (2.4) |
|  | yes | 116 (69.0) |  |
|  | no | 48 (28.6) |  |
|  | **high risk** | **(n=31)*** | n. o. |
|  |  |  | missings |
| Age at start of dialysis, year, median (IQR) | | 55.0 (45.4, 63.8) | 1** (3) |
| Gender | |  | 0 |
|  | male | 22 (71.0) |  |
|  | female | 9 (29.0) |  |
| Body mass index, kg/m^2^, median (IQR) | | 27.3 (24.6, 31.2) | 0 |
| Blood pressure systolic/diastolic, mmHg, median (IQR) | | 140/80 (124/70, 146/82) | 4 (13) |
| Smoking | |  | 9 (29) |
|  | never | 11 (36) |  |
|  | active smoker | 5 (16) |  |
|  | former smoker | 6 (19) |  |
| Diabetes mellitus | |  | 2 (6) |
|  | no | 20 (65) |  |
|  | diabetes mellitus type 1 | 2 (6) |  |
|  | diabetes mellitus type 2 | 7 (23) |  |
|  | diabetes mellitus, other | 0 |  |
| Hypertension | |  | 0 |
|  | yes | 30 (97) |  |
|  | no | 1 (3) |  |
| Hypercholesterolemia | |  | 0 |
|  | yes | 26 (84) |  |
|  | no | 5 (16) |  |

Absolute and relative frequency for categorial data, median and interquartile range (IQR) for metric data.

* missings to add up to 347: Two patients with unavailable categorization according to “Muenster cardiovascular Risk Stratification Score”.

** 16/8/1 patient with unknown date of start of dialysis in the low/intermediate/high risk group (patients with ESRD but not on dialysis)
