# Supplementary material for: A Comparison of Different Algorithms for the Assessment of Cardiovascular Risk in Patients at Waiting List for Kidney Transplantation
Source: PLoS One. 2016 Oct 21;11(10):e0161927. doi: 10.1371/journal.pone.0161927 (PMC5074508; doi:10.1371/journal.pone.0161927)
Supplement: S3 Table — Estimated hazard ratio (HR) for overall survival with 95% confidence interval (CI) and p-value of the likelihood ratio test. For pairwise comparisons, confidence intervals instead of p-values are given (p-value of Wald test ≤ 0.05 if and only if confidence interval does not contain 1). (DOCX) [file pone.0161927.s005.docx]

**S3 Table.** Univariable analysis for overall survival assessing potential risk factors

| **Variable** | **Available cases** | **HR** | **95% CI** | **P*** |
| --- | --- | --- | --- | --- |
| **Gender** | 322 |  |  | **0.378** |
| **female versus male** | 124 vs. 198 | 0.72 | 0.35 to 1.49 |  |
| **Active smoking** | 298 |  |  | **0.084** |
| **yes versus no** | 48 vs. 250 | 2.24 | 0.94 to 5.37 | - |
| **History of smoking** | 274 |  |  | **0.996** |
| **yes versus no** | 50 vs. 224 | 1.00 | 0.29 to 3.47 | - |
| **Hypertension** | 319 |  |  | **0.184** |
| **yes versus no** | 307 vs. 12 | 0.40 | 0.12 to 1.34 |  |
| **Diabetes mellitus** | 313 |  |  | **0.245** |
| **yes versus no** | 51 vs. 262 | 1.79 | 0.71 to 4.52 |  |
| **Hypercholesterinemia** | 314 |  |  | **0.646** |
| **yes versus no** | 202 vs. 112 | 0.83 | 0.37 to 1.84 |  |
| **Hyperlipidemia** | 318 |  |  | **0.671** |
| **yes versus no** | 131 vs. 102 | 1.09 | 0.45 to 2.64 |  |
| **mixed versus no** | 85 vs. 102 | 0.69 | 0.23 to 2.13 |  |
| **Cerebrovascular disease** | 317 |  |  | **0.231** |
| **yes versus no** | 45 vs. 272 | 1.81 | 0.72 to 4.54 |  |
| **Peripheral artery disease** | 313 |  |  | **0.355** |
| **yes versus no** | 42 vs. 271 | 1.58 | 0.62 to 4.06 |  |
| **Coronary artery disease** | 319 |  |  | **0.160** |
| **yes versus no** | 53 vs. 266 | 1.80 | 0.81 to 4.00 |  |
| **Chronic heart failure** | 317 |  |  | **0.255** |
| **yes versus no** | 19 vs. 298 | 1.85 | 0.68 to 5.01 |  |
| **Age at the start of dialysis** | 322 | 1.08 | 1.04 to 1.13 | **<0.001** |
| **Body mass index** | 319 | 0.97 | 0.89 to 1.06 | **0.515** |
| **C-related protein** | 289 | 1.00 | 0.88 to 1.13 | **0.947** |
| **Fibrinogen** | 183 | 1.00 | 0.99 to 1.01 | **0.185** |
| **Dialysis time at time of wait listing** | 313 | 1.01 | 0.99 to 1.03 | **0.524** |

Estimated hazard ratio (HR) for overall survival with 95% confidence interval (CI) and p-value of the likelihood ratio test. For pairwise comparisons, confidence intervals instead of p-values are given (p-value of Wald test ≤ 0.05 if and only if confidence interval does not contain 1).
