# Supplementary material for: A Comparison of Different Algorithms for the Assessment of Cardiovascular Risk in Patients at Waiting List for Kidney Transplantation
Source: PLoS One. 2016 Oct 21;11(10):e0161927. doi: 10.1371/journal.pone.0161927 (PMC5074508; doi:10.1371/journal.pone.0161927)
Supplement: S4 Table — Estimated hazard ratio (HR) for overall survival with 95% confidence interval (CI) and p-value of the likelihood ratio test. For pairwise comparisons, confidence intervals instead of p-values are given (p-value of Wald test ≤ 0.05 if and only if confidence interval does not contain 1). (DOCX) [file pone.0161927.s006.docx]

**S4 Table.** Univariable analysis for overall survival assessing different risk stratification scores

| **Variable** | **Available cases** | **HR** | **95% CI** | **P*** |
| --- | --- | --- | --- | --- |
| ***D-ESC Score*** | 309 | 1.22 | 1.12 to 1.33 | **<0.001** |
| **Graded D-ESC Score** | 309 |  |  | **<0.001** |
| **intermediate versus low risk** | 94 vs. 170 | 3.23 | 1.004 to 10.37 |  |
| **high versus low risk** | 45 vs. 170 | 10.68 | 3.44 to 33.21 |  |
| ***PROCAM Health Score*** | 117 | 1.04 | 0.95 to 1.15 | **0.437** |
| **Graded PROCAM Health Score** | 117 |  |  | **0.953** |
| **intermediate versus low risk** | 31 vs. 70 | 1.47 | 0.13 to 16.53 |  |
| **high versus low risk** | 16 vs. 70 | 1.17 | 0.10 to 13.17 |  |
| ***Framingham Score*** | 118 | 1.04 | 0.90 to 1.20 | **0.635** |
| **“Muenster Risk Stratification”** | 320 |  |  | **0.003** |
| **intermediate versus low risk** | 160 vs. 130 | 6.17 | 1.44 to 26.56 |  |
| **high versus low risk** | 30 vs. 130 | 8.87 | 1.81 to 43.36 |  |

Estimated hazard ratio (HR) for overall survival with 95% confidence interval (CI) and p-value of the likelihood ratio test. For pairwise comparisons, confidence intervals instead of p-values are given (p-value of Wald test ≤ 0.05 if and only if confidence interval does not contain 1).
